# Methyl 2-(5,5-dimethyl-1,3,2-dioxa­borinan-2-yl)-4-nitro­benzoate

**DOI:** 10.1107/S1600536812029650

**Published:** 2012-07-10

**Authors:** S. F. Jenkinson, A. L. Thompson, M. I. Simone

**Affiliations:** a Department of Chemistry, Chemistry Research Laboratory, University of Oxford, Mansfield Road, Oxford OX1 3TA, England; b Department of Chemical Crystallography, Chemistry Research Laboratory, University of Oxford, Mansfield Road, Oxford OX1 3TA, England; c School of Chemistry, University of Sydney, Camperdown 2006, Sydney, Australia

## Abstract

The six-membered boronate ester ring of the title compound, C_13_H_16_BNO_6_, adopts an envelope conformation with the C atom bearing the dimethyl substituents at the flap. The O—B—C—C torsion angles between the boronate group and the benzene ring are 72.5 (2) and 81.0 (2)°. The 4-nitro­benzoate unit adopts a slightly twisted conformation, with dihedral angles between the benzene ring and the nitrate and methyl ester groups of 17.5 (2) and 14.4 (3)°, respectively. In the crystal, inversion-related pairs of mol­ecules show weak π–π stacking inter­actions [centroid–centroid distance = 4.0585 (9) Å and inter­planar spacing = 3.6254 (7) Å].

## Related literature

For use of boronic acids as synthetic inter­mediates, see: Hall (2005 ▶); for their use as sensors in the alcoholic beverage industry, see: Wiskur & Anslyn (2001 ▶) and as saccharide sensors, see: Baxter *et al.* (1990 ▶); Fedorak *et al.* (1989 ▶); Yamamoto *et al.* (1990 ▶); Yasuda *et al.* (1990 ▶). For a review on borolectins, see: Yang *et al.* (2002 ▶, 2004 ▶). For the utilization of boronic acids as enzyme inhibitors, see: Adams *et al.* (1998 ▶); Fevig *et al.* (1996 ▶); Johnson & Houston (2002 ▶); Kettner *et al.* (1990 ▶); Prusoff *et al.* (1993 ▶). For the synthesis of aromatic *ortho*-substituted boronate esters, see: Baudoin *et al.* (2000 ▶); Fang *et al.* (2005 ▶); Ishiyama *et al.* (2010 ▶); Wang *et al.* (2006 ▶).
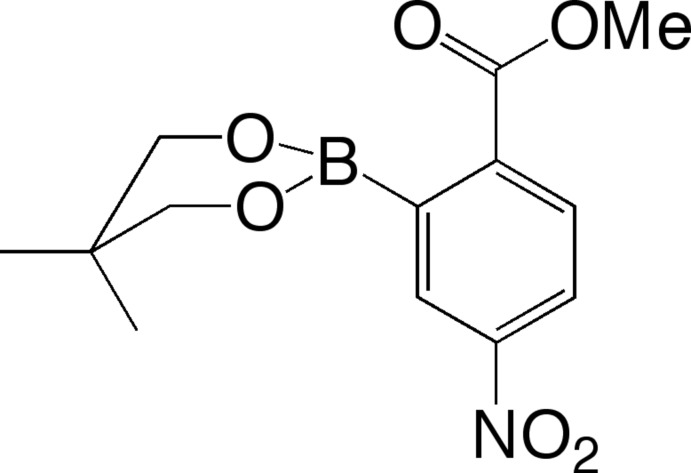


## Experimental

### 

#### Crystal data

C_13_H_16_BNO_6_
*M* *_r_* = 293.08
Monoclinic, 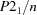

*a* = 12.1774 (3) Å
*b* = 9.7928 (3) Å
*c* = 13.4921 (4) Å
β = 115.4764 (12)°
*V* = 1452.49 (7) Å^3^
*Z* = 4
Mo *K*α radiation
μ = 0.11 mm^−1^
*T* = 150 K
0.25 × 0.20 × 0.15 mm

#### Data collection

Nonius KappaCCD diffractometer
Absorption correction: multi-scan (*DENZO*/*SCALEPACK*; Otwinowski & Minor, 1997 ▶) *T*_min_ = 0.92, *T*_max_ = 0.98
16148 measured reflections
3286 independent reflections
2229 reflections with *I* > 2σ(*I*)
*R*_int_ = 0.043

#### Refinement

*R*[*F* ^2^ > 2σ(*F* ^2^)] = 0.044
*wR*(*F* ^2^) = 0.114
*S* = 0.92
3286 reflections
190 parameters
H-atom parameters constrained
Δρ_max_ = 0.36 e Å^−3^
Δρ_min_ = −0.39 e Å^−3^

### 

Data collection: *COLLECT* (Nonius, 2001 ▶); cell refinement: *DENZO*/*SCALEPACK* (Otwinowski & Minor, 1997 ▶); data reduction: *DENZO*/*SCALEPACK*; program(s) used to solve structure: *SIR92* (Altomare *et al.*, 1994 ▶); program(s) used to refine structure: *CRYSTALS* (Betteridge *et al.*, 2003 ▶); molecular graphics: *CAMERON* (Watkin *et al.*, 1996 ▶); software used to prepare material for publication: *CRYSTALS* and *PLATON* (Spek, 2009 ▶).

## Supplementary Material

Crystal structure: contains datablock(s) global, I. DOI: 10.1107/S1600536812029650/pk2392sup1.cif

Structure factors: contains datablock(s) I. DOI: 10.1107/S1600536812029650/pk2392Isup2.hkl

Supplementary material file. DOI: 10.1107/S1600536812029650/pk2392Isup3.cml

Additional supplementary materials:  crystallographic information; 3D view; checkCIF report

## Figures and Tables

**Fig. 1.**
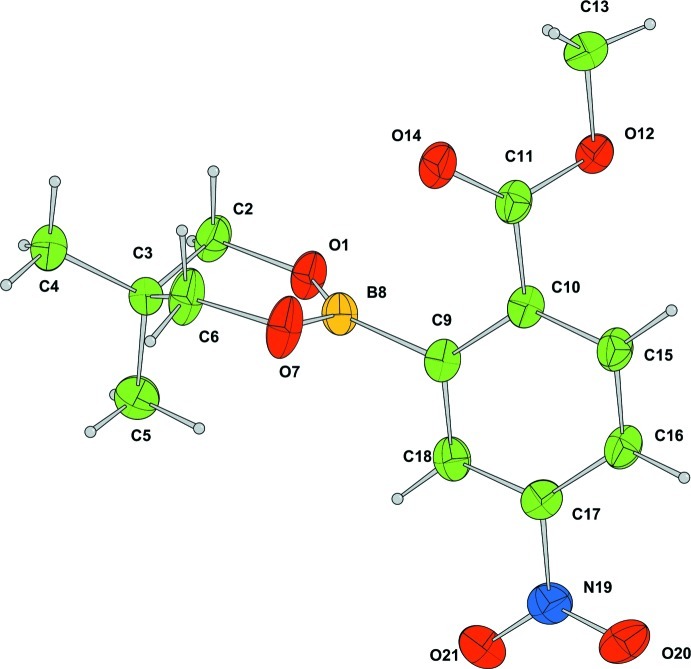
The title compound with displacement ellipsoids drawn at the 50% probability level. Hydrogen atoms are shown as spheres of arbitary radius.

**Fig. 2.**
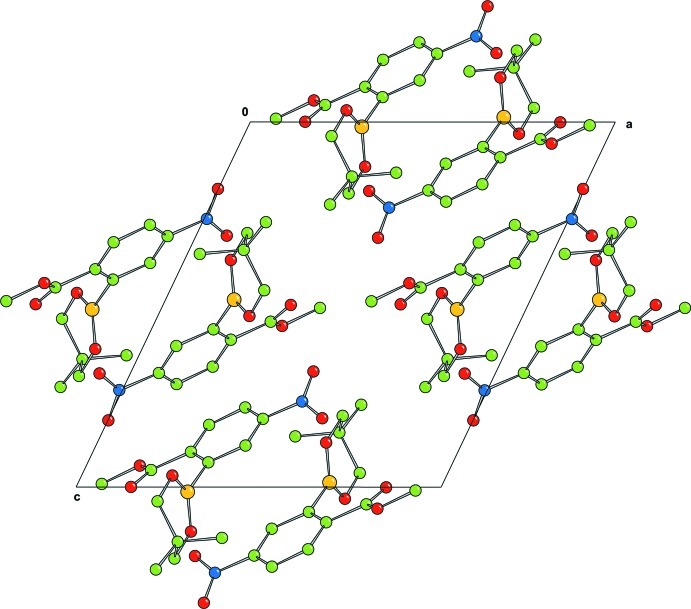
Packing diagram of the title compound projected along the b-axis.

**Fig. 3.**
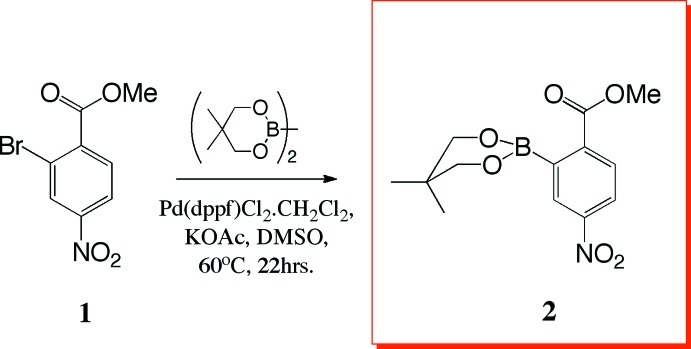
Synthesis of sterically hindered boronate ester 2 from the aryl bromide 1.

## supplementary crystallographic information

### Comment

Boronic acids constitute an important class of synthetic intermediates (Hall, 2005). However, they have found wider applications more recently as sensors of 'gallate-like' compounds in the alcoholic beverage industry (Wiskur & Anslyn, 2001), in the development of saccharide sensors (*in vivo* at neutral pH in aqueous environment) (Baxter *et al.*, 1990; Fedorak *et al.*, 1989; Yamamoto *et al.*, 1990; Yasuda *et al.*, 1990), boronolectins (Yang *et al.*, 2002, 2004), as protease (Fevig *et al.*, 1996; Kettner *et al.*, 1990; Prusoff *et al.*, 1993), glycosidase (Johnson & Houston, 2002) and proteasome inhibitors (Adams *et al.*, 1998).

The synthesis of *ortho*-substituted aromatic esters becomes increasingly difficult as the aromatic ring becomes more substituted (Baudoin *et al.*, 2000; Fang *et al.*, 2005; Ishiyama *et al.*, 2010; Wang *et al.*, 2006). New strategies have recently been developed to circumvent the synthetic obstacles preventing these borylations (Baudoin *et al.*, 2000; Fang *et al.*, 2005; Ishiyama *et al.*, 2010; Wang *et al.*, 2006). Here we report the first successful synthesis and X-ray crystallographic analysis of boronate ester intermediate 2, which is substituted at the *ortho* and *meta* positions by a methyl ester and a nitro group with respect to the boronate ester moiety (Fig. 1).

X-ray crystallography confirmed the structure of the title compound. The six-membered boronate ester ring adopts an envelope type conformation with C3 out of the plane (Fig. 1, 2). The torsion angles between the boronate and the aromatic ring system are 72.5 (2)° and 81.0 (2)°. The 4-nitrobenzoate moiety adopts a slightly twisted conformation with dihedral angles between the benzene ring and the nitrate and methyl ester groups of 17.5 (2)° and 14.4 (3)° respectively. Inversion-related pairs of molecules show π-stacking interactions: Centroid-centroid distance: 4.0585 (9) Å, interplanar spacing: 3.6254 (7) Å. There are no classical hydrogen bonds.

### Experimental

The bromo-nitroester starting material 1 undergoes borylation by stirring with bis(neopentyl glycolato)diboron (1.2 eq.) in the presence of [1,1-bis(diphenylphosphino)ferrocene]dichloropalladium(II) (10 mol%), DMSO and potassium acetate (2.5 eq.) for 22 h at 60°C to afford the corresponding boronate ester 2 in 51% yield (Fig. 3). This reaction worked up to a half gram scale. The purification of the boronate ester 2 was difficult because the bis(neopentyl glycolato)diboron reagent, which was used in excess, proved difficult to completely remove *via* a variety of purification techniques (crystallizations using a range of solvent mixtures and temperatures, flash column chromatography using a range of neutral, acidic and basic solvent mixtures). Methyl 2-(5,5-dimethyl-1,3,2-dioxaborinan-2-yl)-4-nitrobenzoate 2 was isolated as a pale yellow oil which crystallized on standing: m.p. 345–353 K (DCM; it underwent a phase transition over the range 345–351 K, then melted at 351–353 K).

### Refinement

The H atoms were all located in a difference map, but those attached to carbon atoms were repositioned geometrically. The H atoms were initially refined with soft restraints on the bond lengths and angles to regularize their geometry (C—H in the range 0.93–0.98) and *U*_iso_(H) (in the range 1.2–1.5 times *U*_eq_ of the parent atom), after which the positions were refined with riding constraints.

### Crystal data

**Table:**

C_13_H_16_BNO_6_	*F*(000) = 616
*M**_r_* = 293.08	*D*_x_ = 1.340 Mg m^−^^3^
Monoclinic, *P*2_1_/*n*	Mo *K*α radiation, λ = 0.71073 Å
Hall symbol: -P 2yn	Cell parameters from 3373 reflections
*a* = 12.1774 (3) Å	θ = 5–27°
*b* = 9.7928 (3) Å	µ = 0.11 mm^−^^1^
*c* = 13.4921 (4) Å	*T* = 150 K
β = 115.4764 (12)°	Plate, colourless
*V* = 1452.49 (7) Å^3^	0.25 × 0.20 × 0.15 mm
*Z* = 4	

### Data collection

**Table:**

Nonius KappaCCD diffractometer	2229 reflections with *I* > 2σ(*I*)
Graphite monochromator	*R*_int_ = 0.043
ω scans	θ_max_ = 27.5°, θ_min_ = 5.1°
Absorption correction: multi-scan (*DENZO*/*SCALEPACK*; Otwinowski & Minor, 1997)	*h* = −15→15
*T*_min_ = 0.92, *T*_max_ = 0.98	*k* = −12→12
16148 measured reflections	*l* = −17→17
3286 independent reflections	

### Refinement

**Table:**

Refinement on *F*^2^	Primary atom site location: structure-invariant direct methods
Least-squares matrix: full	Hydrogen site location: inferred from neighbouring sites
*R*[*F*^2^ > 2σ(*F*^2^)] = 0.044	H-atom parameters constrained
*wR*(*F*^2^) = 0.114	Method = Modified Sheldrick *w* = 1/[σ^2^(*F*^2^) + ( 0.05*P*)^2^ + 0.66*P*] , where *P* = (max(*F*_o_^2^,0) + 2*F*_c_^2^)/3
*S* = 0.92	(Δ/σ)_max_ = 0.000214
3286 reflections	Δρ_max_ = 0.36 e Å^−^^3^
190 parameters	Δρ_min_ = −0.39 e Å^−^^3^
0 restraints	

### Fractional atomic coordinates and isotropic or equivalent isotropic displacement parameters (Å^2^)

**Table:**

	*x*	*y*	*z*	*U*_iso_*/*U*_eq_	
O1	0.12715 (11)	0.67634 (11)	0.37794 (9)	0.0363	
C2	0.13726 (17)	0.77688 (17)	0.30436 (13)	0.0384	
C3	0.14982 (15)	0.91980 (17)	0.35044 (13)	0.0325	
C4	0.17357 (18)	1.01872 (19)	0.27425 (15)	0.0443	
C5	0.0341 (2)	0.9608 (2)	0.3611 (2)	0.0649	
C6	0.25847 (18)	0.91925 (19)	0.46109 (14)	0.0457	
O7	0.25147 (12)	0.81218 (13)	0.53161 (9)	0.0474	
B8	0.18826 (16)	0.69741 (19)	0.48684 (14)	0.0305	
C9	0.17022 (14)	0.59224 (16)	0.56803 (12)	0.0295	
C10	0.23148 (13)	0.46714 (17)	0.59640 (12)	0.0301	
C11	0.31710 (14)	0.43635 (17)	0.54680 (13)	0.0329	
O12	0.35352 (10)	0.30678 (12)	0.55875 (10)	0.0380	
C13	0.43385 (17)	0.2710 (2)	0.50882 (15)	0.0444	
O14	0.34969 (12)	0.52155 (13)	0.50062 (11)	0.0502	
C15	0.21684 (14)	0.37785 (18)	0.67027 (13)	0.0344	
C16	0.14051 (14)	0.41192 (18)	0.71801 (13)	0.0345	
C17	0.07746 (14)	0.53350 (17)	0.68739 (12)	0.0312	
C18	0.09013 (14)	0.62350 (17)	0.61390 (13)	0.0325	
N19	−0.00773 (13)	0.56838 (15)	0.73455 (12)	0.0385	
O20	0.00100 (11)	0.50649 (14)	0.81687 (10)	0.0451	
O21	−0.08423 (13)	0.65719 (14)	0.68873 (13)	0.0569	
H22	0.2125	0.7563	0.2935	0.0495*	
H21	0.0629	0.7702	0.2345	0.0499*	
H42	0.1825	1.1106	0.3051	0.0710*	
H41	0.2498	0.9921	0.2680	0.0704*	
H43	0.1034	1.0155	0.2010	0.0710*	
H52	0.0436	1.0553	0.3874	0.1049*	
H53	0.0229	0.8990	0.4128	0.1046*	
H51	−0.0345	0.9535	0.2899	0.1051*	
H62	0.2630	1.0067	0.4988	0.0527*	
H61	0.3341	0.9062	0.4495	0.0531*	
H132	0.4574	0.1757	0.5267	0.0723*	
H131	0.5044	0.3321	0.5356	0.0723*	
H133	0.3879	0.2814	0.4298	0.0724*	
H151	0.2617	0.2923	0.6890	0.0415*	
H161	0.1301	0.3524	0.7695	0.0400*	
H181	0.0457	0.7074	0.5971	0.0378*	

### Atomic displacement parameters (Å^2^)

**Table:**

	*U*^11^	*U*^22^	*U*^33^	*U*^12^	*U*^13^	*U*^23^
O1	0.0496 (7)	0.0290 (6)	0.0286 (6)	−0.0096 (5)	0.0151 (5)	0.0004 (5)
C2	0.0548 (10)	0.0329 (9)	0.0298 (8)	−0.0059 (8)	0.0204 (8)	0.0027 (7)
C3	0.0400 (9)	0.0271 (8)	0.0358 (8)	0.0050 (7)	0.0212 (7)	0.0057 (7)
C4	0.0591 (11)	0.0335 (10)	0.0478 (10)	0.0048 (9)	0.0300 (9)	0.0102 (8)
C5	0.0695 (14)	0.0599 (14)	0.0888 (16)	0.0288 (12)	0.0564 (13)	0.0306 (12)
C6	0.0613 (12)	0.0318 (10)	0.0396 (10)	−0.0140 (9)	0.0175 (9)	0.0046 (8)
O7	0.0626 (8)	0.0362 (7)	0.0305 (6)	−0.0196 (6)	0.0078 (6)	0.0041 (5)
B8	0.0313 (9)	0.0274 (10)	0.0290 (9)	−0.0022 (7)	0.0093 (7)	0.0010 (7)
C9	0.0295 (8)	0.0292 (9)	0.0251 (7)	−0.0046 (7)	0.0072 (6)	0.0005 (6)
C10	0.0266 (7)	0.0323 (9)	0.0272 (8)	−0.0035 (7)	0.0077 (6)	0.0025 (7)
C11	0.0292 (8)	0.0347 (10)	0.0317 (8)	−0.0009 (7)	0.0103 (7)	0.0068 (7)
O12	0.0403 (6)	0.0366 (7)	0.0451 (7)	0.0044 (5)	0.0260 (5)	0.0098 (5)
C13	0.0498 (10)	0.0436 (11)	0.0531 (11)	0.0030 (9)	0.0348 (9)	0.0049 (9)
O14	0.0525 (8)	0.0402 (8)	0.0727 (9)	0.0043 (6)	0.0408 (7)	0.0197 (7)
C15	0.0308 (8)	0.0362 (10)	0.0345 (9)	0.0029 (7)	0.0124 (7)	0.0107 (7)
C16	0.0341 (8)	0.0385 (10)	0.0293 (8)	−0.0012 (7)	0.0120 (7)	0.0070 (7)
C17	0.0310 (8)	0.0345 (9)	0.0262 (8)	−0.0051 (7)	0.0104 (6)	−0.0046 (7)
C18	0.0345 (8)	0.0268 (9)	0.0308 (8)	−0.0030 (7)	0.0091 (7)	−0.0018 (7)
N19	0.0441 (8)	0.0347 (8)	0.0399 (8)	−0.0064 (7)	0.0211 (7)	−0.0089 (7)
O20	0.0517 (7)	0.0552 (8)	0.0336 (6)	−0.0089 (6)	0.0233 (6)	−0.0066 (6)
O21	0.0658 (9)	0.0415 (8)	0.0790 (10)	0.0151 (7)	0.0459 (8)	0.0069 (7)

### Geometric parameters (Å, º)

**Table:**

O1—C2	1.4406 (18)	C9—C10	1.399 (2)
O1—B8	1.347 (2)	C9—C18	1.395 (2)
C2—C3	1.512 (2)	C10—C11	1.492 (2)
C2—H22	1.009	C10—C15	1.394 (2)
C2—H21	0.989	C11—O12	1.331 (2)
C3—C4	1.528 (2)	C11—O14	1.2061 (19)
C3—C5	1.531 (2)	O12—C13	1.4492 (19)
C3—C6	1.510 (2)	C13—H132	0.976
C4—H42	0.977	C13—H131	0.979
C4—H41	1.002	C13—H133	0.974
C4—H43	0.990	C15—C16	1.380 (2)
C5—H52	0.980	C15—H151	0.972
C5—H53	0.977	C16—C17	1.380 (2)
C5—H51	0.967	C16—H161	0.956
C6—O7	1.443 (2)	C17—C18	1.384 (2)
C6—H62	0.986	C17—N19	1.471 (2)
C6—H61	1.006	C18—H181	0.955
O7—B8	1.349 (2)	N19—O20	1.2291 (18)
B8—C9	1.586 (2)	N19—O21	1.2292 (19)
			
C2—O1—B8	118.43 (13)	O7—B8—C9	116.97 (14)
O1—C2—C3	111.88 (13)	O1—B8—C9	118.56 (14)
O1—C2—H22	108.4	B8—C9—C10	122.79 (14)
C3—C2—H22	107.9	B8—C9—C18	119.65 (14)
O1—C2—H21	107.3	C10—C9—C18	117.56 (14)
C3—C2—H21	110.0	C9—C10—C11	116.57 (14)
H22—C2—H21	111.4	C9—C10—C15	121.85 (15)
C2—C3—C4	108.97 (13)	C11—C10—C15	121.53 (15)
C2—C3—C5	110.29 (16)	C10—C11—O12	113.52 (13)
C4—C3—C5	110.20 (15)	C10—C11—O14	122.73 (16)
C2—C3—C6	107.08 (14)	O12—C11—O14	123.75 (15)
C4—C3—C6	109.18 (14)	C11—O12—C13	115.50 (13)
C5—C3—C6	111.04 (16)	O12—C13—H132	107.6
C3—C4—H42	108.4	O12—C13—H131	110.0
C3—C4—H41	110.0	H132—C13—H131	112.0
H42—C4—H41	109.7	O12—C13—H133	107.4
C3—C4—H43	108.6	H132—C13—H133	109.9
H42—C4—H43	110.1	H131—C13—H133	109.8
H41—C4—H43	110.0	C10—C15—C16	120.07 (15)
C3—C5—H52	108.1	C10—C15—H151	119.8
C3—C5—H53	108.7	C16—C15—H151	120.2
H52—C5—H53	110.9	C15—C16—C17	117.93 (15)
C3—C5—H51	108.9	C15—C16—H161	121.2
H52—C5—H51	110.2	C17—C16—H161	120.9
H53—C5—H51	109.8	C16—C17—C18	123.00 (15)
C3—C6—O7	112.30 (14)	C16—C17—N19	118.41 (14)
C3—C6—H62	109.8	C18—C17—N19	118.59 (15)
O7—C6—H62	107.3	C9—C18—C17	119.52 (15)
C3—C6—H61	108.5	C9—C18—H181	121.0
O7—C6—H61	109.1	C17—C18—H181	119.4
H62—C6—H61	109.8	C17—N19—O20	118.24 (14)
C6—O7—B8	119.62 (13)	C17—N19—O21	118.01 (14)
O7—B8—O1	123.85 (15)	O20—N19—O21	123.75 (15)

### Hydrogen-bond geometry (Å, º)

**Table:**

*D*—H···*A*	*D*—H	H···*A*	*D*···*A*	*D*—H···*A*
C4—H42···O21^i^	0.98	2.59	3.460 (3)	149
C13—H132···O20^ii^	0.98	2.56	3.356 (3)	139
C13—H131···O14^iii^	0.98	2.49	3.373 (3)	150
C16—H161···O7^ii^	0.96	2.47	3.205 (3)	134

Symmetry codes: (i) −*x*, −*y*+2, −*z*+1; (ii) −*x*+1/2, *y*−1/2, −*z*+3/2; (iii) −*x*+1, −*y*+1, −*z*+1.
